# Spectral Characterization and Unmixing of Intrinsic Contrast in Intact Normal and Diseased Gastric Tissues Using Hyperspectral Two-Photon Microscopy

**DOI:** 10.1371/journal.pone.0019925

**Published:** 2011-05-16

**Authors:** Lauren E. Grosberg, Andrew J. Radosevich, Samuel Asfaha, Timothy C. Wang, Elizabeth M. C. Hillman

**Affiliations:** 1 Laboratory for Functional Optical Imaging, Departments of Biomedical Engineering and Radiology, Columbia University, New York, New York, United States of America; 2 Division of Digestive and Liver Diseases, Department of Medicine, Columbia University Medical Center, New York, New York, United States of America; University of California, Merced, United States of America

## Abstract

**Background:**

Living tissues contain a range of intrinsic fluorophores and sources of second harmonic generation which provide contrast that can be exploited for fresh tissue imaging. Microscopic imaging of fresh tissue samples can circumvent the cost and time associated with conventional histology. Further, intrinsic contrast can provide rich information about a tissue's composition, structure and function, and opens the potential for in-vivo imaging without the need for contrast agents.

**Methodology/Principal Findings:**

In this study, we used hyperspectral two-photon microscopy to explore the characteristics of both normal and diseased gastrointestinal (GI) tissues, relying only on their endogenous fluorescence and second harmonic generation to provide contrast. We obtained hyperspectral data at subcellular resolution by acquiring images over a range of two-photon excitation wavelengths, and found excitation spectral signatures of specific tissue types based on our ability to clearly visualize morphology. We present the two-photon excitation spectral properties of four major tissue types that are present throughout the GI tract: epithelium, lamina propria, collagen, and lymphatic tissue. Using these four excitation signatures as basis spectra, linear unmixing strategies were applied to hyperspectral data sets of both normal and neoplastic tissue acquired in the colon and small intestine. Our results show that hyperspectral unmixing with excitation spectra allows segmentation, showing promise for blind identification of tissue types within a field of view, analogous to specific staining in conventional histology. The intrinsic spectral signatures of these tissue types provide information relating to their biochemical composition.

**Conclusions/Significance:**

These results suggest hyperspectral two-photon microscopy could provide an alternative to conventional histology either for in-situ imaging, or intraoperative ‘instant histology’ of fresh tissue biopsies.

## Introduction

Biological tissue contains many molecules with intrinsic contrast, either from endogenous absorption and fluorescence or from second harmonic generation (SHG). Harnessing intrinsic contrast enables imaging of intact tissues with subcellular resolution, with the added benefit of providing biochemical information without the need for tissue fixing, sectioning, or staining. Two-photon microscopy is an ideal tool for studying intrinsic fluorescence and SHG in fresh tissues because it can excite endogenous fluorophores in the ultraviolet-to-blue range where their cross-section is often highest, using lower energy longer wavelength light [1]. Exciting with longer wavelengths reduces photodamage to tissue outside the focal plane and increases the achievable penetration depth into scattering media. The depth-sectioning capabilities of two-photon microscopy also allow 3-dimensional (3D) visualization of intact tissues to depths of 200–500 microns, for appreciation of 3D structures without the need for tissue slicing or co-registration [2]. Additionally, the tunable lasers often used in two-photon microscopy enable automated image acquisition at multiple excitation wavelengths to create rich 4D data sets with both structural detail and spectroscopic information [3]. Acquiring spectroscopic data at high resolution adds functional and biochemical knowledge of tissues on a sub-micron scale to already valuable microscopic visualization of 3D morphology.

We recently demonstrated that we can delineate and quantify the relative concentrations of multiple intrinsic fluorophores in living mouse skin using our hyperspectral two-photon microscopy approach [3]. The purpose of the current study was to apply hyperspectral microscopy to healthy and diseased gastrointestinal (GI) tissues in order to visualize morphology of fresh tissue, characterize the intrinsic contrast within the gastric mucosa, and determine the spectral signatures of the major tissue components that make up GI tissues. Spectroscopic studies in bulk tissues have suggested that the most common sources of fluorescence in GI tissues are reduced nicotinamide adenine dinucleotide (NADH), flavin adenine dinucleotide (FAD), collagen, porphyrins, tryptophan and phospholipids [4]. However, such bulk tissue studies capture all of these fluorescent emissions in together, with no knowledge of the cellular compartments from which they originate, making measured spectra difficult to separate and interpret [5], [6]. Our combined high resolution/spectroscopic technique is therefore useful because it uses knowledge about the structure of tissue to isolate the fluorescence signal from small volumes that contain fewer fluorescent species than bulk tissue. Our results demonstrate the value of hyperspectral two-photon for imaging gastric mucosa and also affirm that two-photon microscopy is well suited to resolving subcellular morphology in fresh GI tissues using only endogenous contrast [7].

Several recent studies have shown significant progress towards the development of techniques such as confocal microendoscopy [8], [9], [10], allowing acquisition of microscopic images of in-situ tissues including in the GI tract. Additional studies have also demonstrated the promise of two-photon microscopy for imaging cancer in skin, lung, and bladder [11], [12], [13]. These studies suggest that our hyperspectral microscopy technique has significant potential for clinical translation, either for in-situ intraoperative, or real-time bedside ‘instant histology’.

## Methods

### Instrumentation and imaging techniques

Images were acquired using our custom built two-photon microscope. A tunable Ti:Sapphire laser (MaiTai XF, Spectra Physics) provides excitation light that is focused with an Olympus XLUMPlanFl 20×/0.95 W objective mounted on a z-translation stage (M-112.1DG, PI). Two galvanometric mirrors (Cambridge Technologies) are used to steer the beam in an x-y raster scan. Fluorescence is simultaneously collected by three spectrally resolved photomultiplier detectors (blue: 350–505 nm, green: 505–560 nm, and red: 560–650 nm emission ranges, R3896 Hamamatsu). Single frame images displayed in the paper are 400×400 pixels, and were acquired at 2.5 frames/s. Custom software (written as a Matlab™ graphical user interface) controls the laser power and wavelength, as well as all translational motion, image acquisition, gain and amplifier settings, image display, and online processing. The software allows image acquisition while synchronously tuning the wavelength of the Ti:Sapphire laser and monitoring its power. Average power at the sample was 1–3 mW and varies with wavelength. For hyperspectral acquisition, an image was obtained at each step as the laser was tuned from 710–920 nm in 5 nm increments. A full wavelength scan can be acquired in 60 s. For basic visualization of acquired images, red-green-blue (RGB) merges were created using data acquired with the three emission channels, with each channel scaled to its maximum after thresholding out the highest 0.06% of pixels.

### Animal models

All procedures followed were reviewed and approved by the Columbia University Institutional Animal Care and Use Committee.

Freshly excised tissues from a total of 15 (10 normal and 5 transgenic) recently euthanized mice were used for imaging and spectral analysis. The animals were euthanized with an overdose of isoflurane, dissected, and then selected GI tissues were dissected out and imaged. Tissue was bathed in saline prior to imaging, and all imaging occurred within four hours of euthanasia. In some cases, images were acquired from the outside of the gastric lumen, with the tissue still intact in the body. In other cases, a piece of GI tissue was removed, opened flat, gently rinsed with saline and imaged on a recessed microscope slide. Tissues were then processed, sectioned and stained with hematoxylin and eosin (H&E) for comparative histology.

#### Cancer model

A transgenic mouse model (Male C57BL/6J-APC *Min*/+) that spontaneously develops malignant polyps in the small intestine was used for comparison of neoplastic to normal tissue. A total of 5 transgenic mice were imaged at 15–20 weeks of age, a stage at which adenomas should be developed. Data from one of these mice is shown in this paper.

## Results

### Basic morphology of the lower gastrointestinal tract

**Figure 1:**
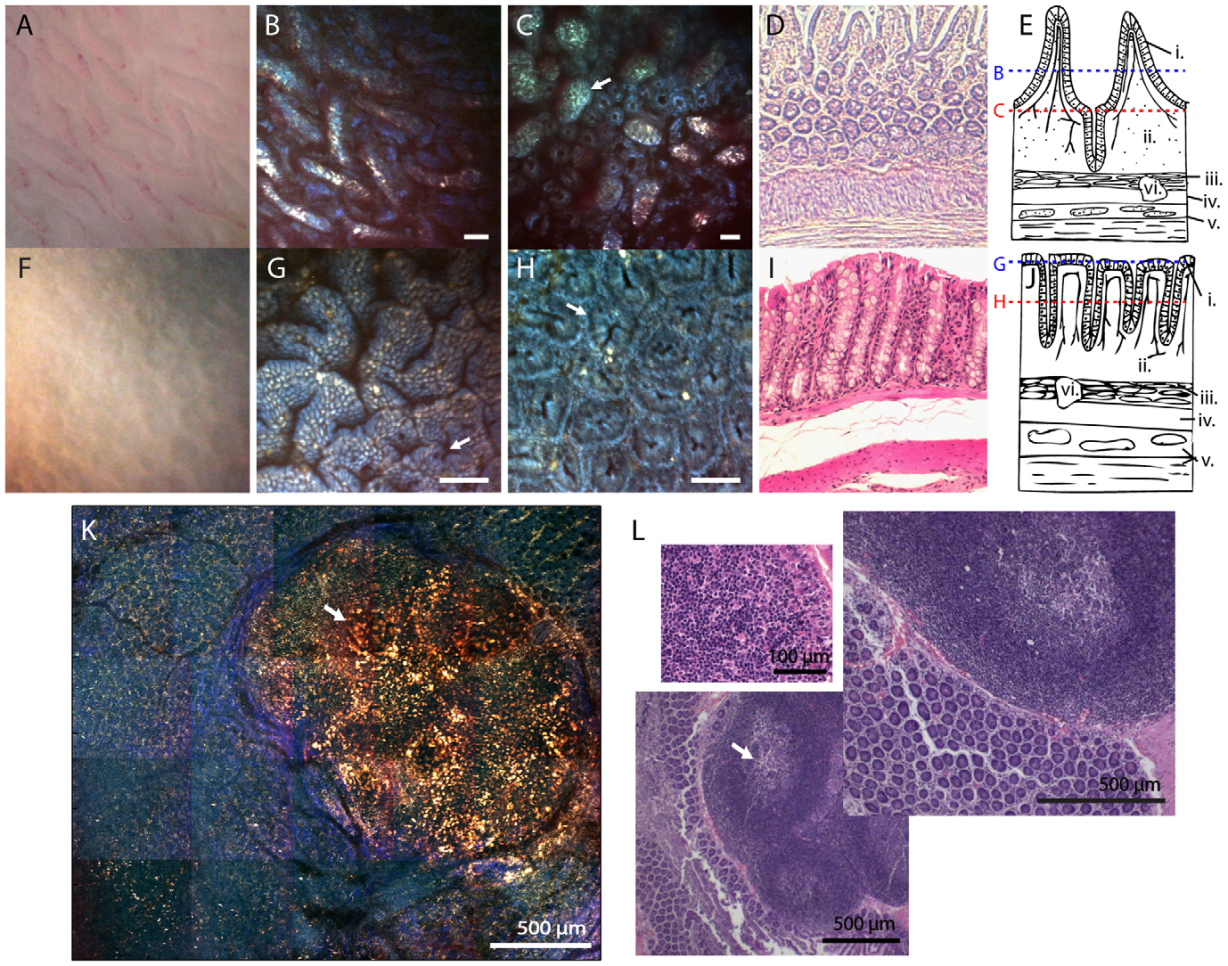
Comparison of small intestine and colon using white light imaging, two-photon microscopy and conventional H&E histology. Two-photon images are shown as RGB merges normalized to the thresholded maximum of each channel. Scale bar is 50 µm except where otherwise noted. (A) An en-face white light image of fresh tissue from normal small intestine at 20× magnification provides a 2D view of the villi laying flat. (B) The same field of view as in A acquired using two-photon microscopy of intrinsic contrast with 740 nm excitation. It is possible to distinguish between the epithelial layer that covers the villi (blue) and the core of each villus (yellow). (C) A two-photon image of a deeper layer of the small intestine reveals intestinal glands, or crypts of Lieberkühn at the base of the villi. Arrow points to villi. (D) Cross-sectional H&E histology of the small intestine showing the layers of the mucosa. B was acquired at the villi level and C was acquired at the crypt level. (E) Schematic morphology of the small intestine. Epithelium (i.) covers the villi and forms the crypts. Lamina propria (ii.) extends into the core of the villi and contains a vascular network. The remaining layers are the muscularis mucosa (iii.) submucosa (iv) and the muscularis externa (v). Lymphoid aggregates or Peyer's patches of varying size (vi.) are found in the submucosal layers. (F) White light image of fresh, normal colon tissue at 20× magnification. (G) A two-photon image of intrinsic contrast showing the folds in the superficial epithelial layer of the colon. Arrow points to the openings to the crypts of Lieberkühn. (H) A deeper two-photon image of the same piece of colon tissue revealing densely packed crypts 80 µm below the epithelial layer shown in E. Nuclei are visible as black dots circling the outside of the glands (arrow). (I) Cross-sectional H&E histology of the colon showing that the crypts extend through the full thickness of the lamina propria. (J) Schematic morphology of the colon. Epithelium (i.) lines the mucosa and extends into the lamina propria (ii.) to form the crypts. The remaining layers are the muscularis mucosa (iii.) submucosa (iv) and the muscularis externa (v). As in the small intestine lymphoid aggregates or Peyer's patches of varying size (vi.) are found in the submucosal layers. (K) Two photon image of a Peyer's patch found in the small intestine. Scale bar is 500 µm. Arrow points to a germinal center of the lymphoid aggregate. L Shows a range of H&E stained Peyer's patches at similar magnification to K (inset shows 40× fine detail of aggregate).

The gastric mucosa collectively includes the epithelium, lamina propria, and muscularis mucosa. Figure 1 shows a schematic diagram of the general organization of the small intestine and colon. In the small intestine, projections called villi increase surface area for more efficient digestion and absorption. The lamina propria extends to form the core of the villi, and a continuous layer of epithelium forms a barrier between this core and the contents of the intestinal lumen (Figure 1E). Epithelial tissue is comprised of a single layer of columnar epithelial cells, which generally function to absorb nutrients and fluid. Specialized goblet cells within the epithelium secrete mucin as a lubricant and chemical barrier. At the base of the villi, epithelial tissue dives into the lamina propria layer to form crypts of Lieberkühn, which are intestinal glands located throughout the GI tract. The lamina propria of the small intestine is made up of loose connective tissue including lymphocytes, fibroblasts, plasma cells, other lymphatic tissue, and a network of blood capillaries [14]. Smooth muscles cells are present in the villi alongside central lymphatic capillaries called lacteals. In the colon (Figure 1J), lamina propria is much the same as in the small intestine except for the absence of lymphatic vessels and more well developed lymphoid tissue. In addition, there are no villi but a larger number of secreting goblet cells. Crypts of Lieberkühn are more densely packed. Lymphoid aggregates, or Peyer's patches, are present in both the small and large intestines and are important for the GI's immune response (Figure 1L). Peyer's patches are comprised primarily of lymphocytes and contain a network of connective tissues including type-IV collagen, laminin, and fibronectin [15].

### Comparison of morphology

In Figure 1, two-photon microscopy of intrinsic contrast is used to visualize the morphology of normal small intestine and colon, and is shown alongside high magnification white light imaging and conventional histology. White light images were acquired from fresh tissue using a digital camera focused through the eyepiece and objective lens of our two-photon microscope. Although the white light images alone show contours of the tissue, they cannot resolve cellular morphology (Figure 1A & 1F). The two-photon images (acquired with an excitation wavelength of 740 nm) can distinguish not only individual cells, but subcellular structures such as nuclei, which appear as dark circles (arrow in Figure 1H). Figure 1B shows villi laying flat on the inner surface of the small intestine, while Figure 1C shows the deeper glandular level of the small intestine. Circular crypts at the base of the villi can be seen. Figure 1F shows the superficial epithelial layer of the colon, while Figure 1G shows a deeper layer with tightly packed crypts. Figure 1K shows a mosaic of images spanning a 2.5 mm wide piece of tissue that included a Peyer's patch. While in the single images composing this mosaic, individual crypts can be clearly seen in the tissues surrounding the patch, at the ensemble scale, the undulations of the mucosa can be seen, along with the structural changes that the growing Peyer's patch has imposed on its surroundings. Corresponding histology images of Peyer's patches are shown in Figure 1L.

All of these images were acquired with an excitation wavelength of 740 nm, and images shown are red-green-blue merges of data from our three emission channels, scaled to the maximum in each channel for each image. However it is clear from these images, that the epithelial tissue composing the outer layers of the villi, and the crypts of both the small intestine and colon have a distinctive blue color. Another common feature are yellow-white bright spots that can be observed within the villi cores and laminar propria. Particularly around the Peyer's patch, pure blue-colored filamentous structures can be seen. While these ‘color codings’ are relatively simple to pick out of the images, they are rather qualitative and subjective. They also raise many questions which we will directly address below with our hyperspectral microscopy technique, namely; 1) what substances are causing these distinctive colors? and 2) can these colors be used to isolate and identify key structures in each image (such as crypts)?

The images in Figure 1 also highlight one of the additional major benefits of two-photon imaging on fresh tissue; 3D sectioning. Conventionally, histology is used to visualize morphology, which requires destructive physical slicing for sectioning and often distortion and a lack of co-registration between slices. The two-photon images in Figures 1G and H were acquired in the same piece of tissue, simply by moving the microscope objective along the z direction. The relative depths of the two-photon images in Figures 1B, C, G and H are indicated by the dashed lines in Figures 1E and J. Two supplemental movies are also provided showing intrinsic contrast, depth-resolved stacks acquired in the small intestine. Video S1 shows a stack from the tips of the villi to midway down their extent through to a depth of 100 µm. Video S2 shows a stack through the small intestine spanning from the outer muscularis mucosa through the glandular layer to the base of the villi, through to a depth of 170 µm.

### Excitation wavelength dependence of intrinsic contrast

**Figure 2:**
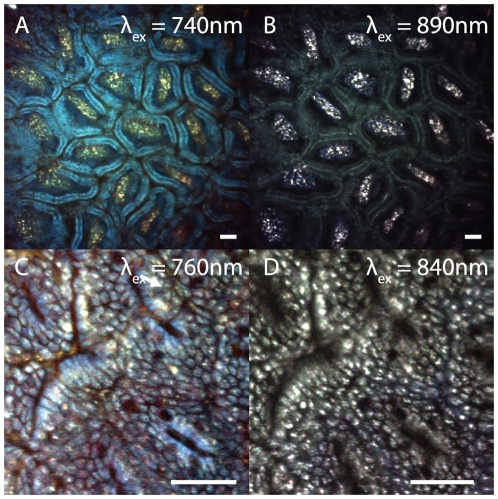
The effects of varying two-photon excitation wavelength. The structures visible in the same field of view depend on the excitation wavelength that is used. The images shown are averages over a 25 nm range at 5 nm steps with center wavelengths reported in the figure. (A) Image of the villi in the small intestine at 740 nm. The epithelial layer around the villi appears blue. (B) At λ_ex_ = 890 nm, the centers of the villi show the strongest contrast while the outer epithelial layer appears green. (C) Layer of epithelium closest to the lumen in the colon. Colon folds and individual cells are visible at 740 nm. (D) At higher excitation wavelengths, bright patches of fluorescence are more prominent within individual cells. Images are scaled RGB merges, so it should be noted that the relative color mix in each image is determined only by the particular structures in each image. Scale bars, 50 µm.

As stated above, the two-photon images shown in Figure 1 were acquired using an excitation wavelength of 740 nm, and while tissue components had fairly distinctive ‘colors’, the origins of these signals were difficult to quantify. However, if the excitation wavelength is varied, different structures within the image contribute varying amounts of signal to the red, green, and blue emission channels, thereby revealing different information about the origin of each tissue type's fluorescence. Figure 2 shows two-photon images acquired at the same location but with different excitation wavelengths. Figures 2A & B show a region of the small intestine in a plane that transects the villi. At 740 nm excitation, the outer layers of the villi appear blue like the crypts in the colon (Fig. 2A). However, at 890 nm, the same region exhibits dark green fluorescence (Figure 2B). The epithelial surface of the colon in Figs. 2C & D can be seen to be composed of individual cells which have regions that appear blue at 740 nm, but which show different intracellular structures at 840 nm excitation. A wavelength scan at the glandular level of the small intestine is included as Video S3.

### Image-guided two-photon spectroscopy

We can harness these excitation-dependent spectral changes using hyperspectral wavelength scans, whereby images are acquired at a series of excitation wavelengths, generating an excitation spectrum (for our 3 emission bands) at every pixel in the image. Rather than qualitatively comparing the emission of different tissue types, we can then use this hyperspectral data to determine the specific excitation-emission spectral signatures of the major tissue types that we observe in our two-photon images.

Spectral signatures were therefore extracted from small regions within each data set that corresponded to particular common tissue types/species; epithelium, laminar propria, collagen and lymphatic tissue. Choice of these regions was guided by our knowledge of the structures and organization of the colon and our ability to visualize common morphologies as shown in Figures 1 and 2. ‘Epithelium’ was chosen from the outer layers of the villi in the small intestine (blue in Figure 1B) and the regions making up the crypts in the colon (Figure 1G). ‘Lamina propria’ was most easily distinguishable beneath the epithelium in the villi, and was therefore chosen from small intestine only. We limited our definition of lamina propria to the small intestine both because segmentation was clearer and because lamina propria in the large intestine contains additional connective tissue which we captured in the collagen component. ‘Collagen’ signatures were selected from regions that looked fibrous and exhibited bright blue SHG at longer excitation wavelengths. ‘Lymphatic tissue’ signatures were chosen from Peyer's patches found in the small intestine, which are composed primarily of lymphocytes.

Basis spectra were calculated by averaging the values of the pixels in each selected region at each excitation wavelength and for all three emission channels. This process was repeated for various image sites (2–6) within mice and in different mice (2–4) for these specific tissues in order to obtain their average ‘basis spectra’. The overall signal amplitude of extracted spectra varied depending on imaging depth; however, the shapes of the excitation spectra were quite consistent. We therefore normalized each basis spectrum set (corresponding to excitation spectra for the blue, green and red emission channels) to the maximum of the blue emission channel's excitation spectrum prior to calculating the average basis spectra for each tissue type across mice. This normalization preserved the relative amplitudes of the blue, green and red emission bands.

Raw basis spectra extracted in this way are not calibrated to account for wavelength-dependent effects originating from our system. As shown in Equations 1 and 2 below, in addition to the properties of the sample, variations in two-photon emission can also result from wavelength-dependent changes in laser power and pulse width, dispersion through our system's optical elements, numerical aperture the and excitation wavelength itself. The two-photon fluorescence collected from the sample is given by Equations 1 and 2 [16]:
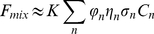
(1)

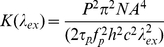
(2)where *F_mix_* is the detected fluorescence, which is assumed to be a linear sum of the fluorescence of each species, *C* is the concentration of the fluorophore, *η* is its quantum efficiency, *ϕ* is the collection efficiency of the detector, and *σ* is its two-photon absorption cross section. *K* is a multiplicative factor where *P* is the laser power, *τ_p_* is the excitation pulse width, *f_p_* is the laser pulse repetition rate, *NA* is the numerical aperture of the lens, *h* is Planck's constant, *c* is the speed of light, and *λ_ex_* is the excitation wavelength.

Based on these equations, *K* is a wavelength-dependant factor which is partially dependent on the arrangement of optical components within any two-photon instrument. As such, K will vary between different systems. However, for our system, we have found that K is generally repeatable and consistent between multiple scans.

In order to more clearly visualize the extracted two-photon excitation-emission basis spectra of the selected tissues, and thereby better understand their composition, it was necessary to determine *K*. We therefore needed a standard fluorophore either with a known two-photon excitation spectrum, or one with an approximately flat excitation spectrum across our excitation range. We chose to use a pure solution of FAD (Sigma Aldrich F6625) as this reference fluorophore, since the absolute one-photon and two-photon excitation cross sections of FAD have been reported as relatively flat for excitation wavelengths between 355–460 nm/710–900 nm respectively [3], [17].

To obtain an FAD reference spectrum, we measured the two-photon fluorescence from the green emission channel of our system (505–560 nm) while scanning a small well beneath a cover glass filled with a 400 µM solution of FAD (Sigma Aldrich F6625) in phosphate buffered saline (PBS). Assuming that *η*, *ϕ* and *σ* for FAD are approximately excitation wavelength-invariant, means that this green emission channel measurement is equivalent to K multiplied by a single scalar value. The basis spectra extracted from our wavelength scans of GI tissues were therefore divided by this measured FAD excitation spectrum to remove the effect of *K* from all three emission channels of our data. The uncalibrated basis spectra are included as Figure S1 and the calibration process is illustrated in Figure S2. While this approach adds the small effect of the excitation-wavelength dependence of FAD fluorescence to the spectra shown, the calibration removes much larger enveloping from systematic effects and allows comparison of our spectra to each other, and to one-photon excitation spectra.

**Figure 3:**
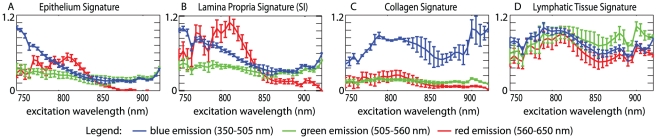
Mean spectral signatures extracted from four tissue types, after spectral calibration via normalization by in-vitro FAD spectra. Error bars are one standard deviation above and below each data point. (A) Epithelium: n = 6 (4 mice, 6 sites) (B) Lamina propria: n = 3 (2 mice, 3 sites) Lamina propria signatures were all taken from samples in the small intestine to minimize collagen overlap. (C) Collagen: n = 4 (2 mice 4 sites) (D) Lymphatic tissue: n = 4 (2 mice 4 sites).

In order to account for the wavelength-dependent spectral response of our PMT detectors, their gains, and emission filter efficiency and bandwidth differences between emission channels, scaling factors were also applied to the green and red channels. These scaling factors were determined using back-illumination of the system with 3 narrow-band filtered LEDs adjusted to have identical powers within the blue, green and red emission channels' bandwidths. Figure 3 shows the final extracted basis spectral signatures, after these spectral calibrations, corresponding to the four selected tissue types; epithelial tissue, lamina propria, collagen and lymphatic tissue.

### Origin of intrinsic contrast

These calibrated spectra allow interpretation of the biochemical constituents of each of the selected tissue types. Figure S2 includes two-photon excitation-emission spectra of a range of pure in-vitro samples of known intrinsic fluorophores, specifically FAD, NADH, zinc protoporphyrin and phospholipid, as well as whole blood. Based on these spectra, and reported 1- and 2-photon spectra of intrinsic fluorophores [1], [18] we hypothesize that the primary contributor to the epithelial fluorescence signature is most likely NADH, given its strong emission in the blue channel when excited with wavelengths <800 nm. The flat green emission for all excitation wavelengths suggests FAD also contributes to the epithelial signature. Epithelial cells are constantly regenerating, therefore it is reasonable to expect NADH and FAD will be major sources of autofluorescence [19]. Lamina propria likely also contains NADH and FAD, however the different excitation patterns in each emission channel suggest there are multiple contributors to its spectral signature. Since the lamina propria contains the GI capillary network, we suspect that porphyrin fluorescence in blood is resulting in the peak in red emission at lower excitation wavelengths. The lymphatic tissue signal is similar in all three emission channels, suggesting that its origin is a single fluorophore with broad excitation and emission. Few intrinsic fluorophores have strong red emission [20], therefore we suspect the fluorescence originates from phospholipids, known to be a significant component of lymphocytes [21]. Almost the entire collagen signal is in the blue emission channel, since the major signal that we detect from collagen is due to SHG. SHG generates narrow-band light at half the excitation wavelength, with higher signal coming from longer wavelengths. At wavelengths lower than around 780 nm, the SHG light generated at <390 nm will be heavily attenuated in tissue, and will not transmit well through our system's glass lenses and filters.

Hyperspectral two-photon microscopy can therefore provide much enhanced analysis of the underlying composition of tissues based not only at their apparent emission ‘colors’ in single excitation images, but on their interesting and complex excitation and emission spectra.

### Hyperspectral Unmixing

Extracting spectra from small regions and averaging over multiple samples does not necessarily demonstrate that a given signature is wholly unique to a particular tissue type. Hyperspectral unmixing offers a way of delineating individual regions within a hyperspectral data set, based on their spectral signatures [3]. Performing unmixing allows all regions with similar spectra, and by extension similar composition, to be separated from other structures within the image in an analogous way to specific chemical or histological staining. Well-delineated unmixing with small residuals further demonstrates the purity of the extracted basis spectra as signatures of each tissue type.

Hyperspectral unmixing assumes that the signal detected in each voxel is a linear sum of the fluorescence and SHG signals from its constituents, scaled by their relative concentrations (Equations 1). Unmixing is therefore performed pixel-by-pixel using a non-negative least squares fit (lsqnonneg in Matlab™) to derive four coefficients per pixel corresponding to the relative contribution of each basis spectrum to the overall spectrum of the pixel. Visualizing each of these coefficients as an image reveals the relative amounts of each constituent within the image. As we showed in a previous study [3], it is not necessary to calibrate for the wavelength-dependent factor K during unmixing, since it should affect all basis spectra and measured hyperspectral data in the same way. We therefore did not perform spectral calibrations on the basis spectra before unmixing to avoid the introduction of unnecessary systematic effects or rounding errors. To demonstrate the robustness of our spectral signatures, basis spectra used for unmixing were not derived from these same data sets that underwent unmixing.

**Figure 4:**
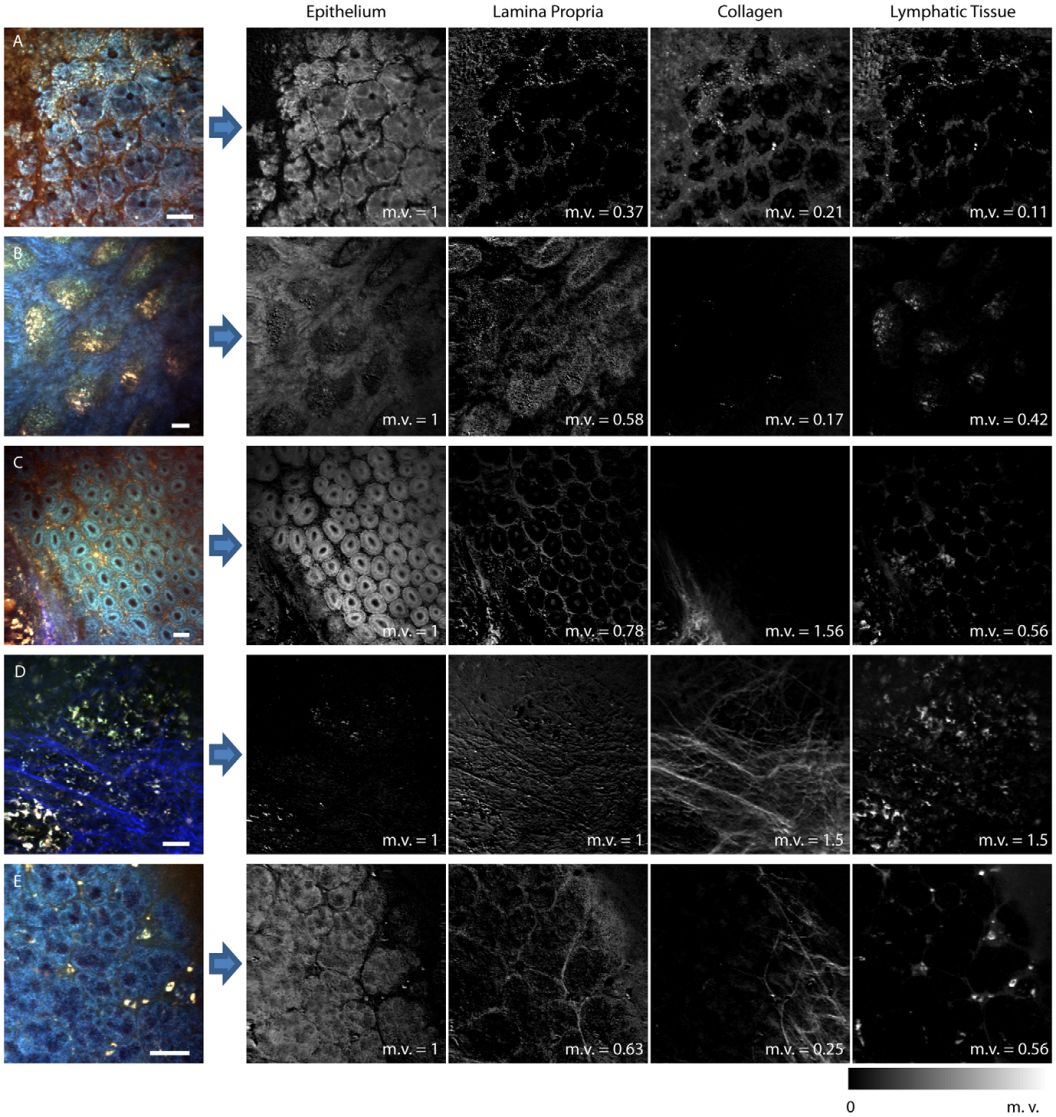
Hyperspectral data unmixing of images of gastric tissues using averaged basis spectra. RGB images are at 740 nm excitation, except for D is at 890 nm. Grayscale images are scaled from 0 to the maximum value (m.v.) reported in each image. (A) Image of glandular later of normal colon shows crypts and the surface epithelium. (B) Image of the villi in the small intestine. Epithelial tissue, lamina propria, and lymphatic tissue can be seen, although very little collagen is detected. (C) Image of the glandular level of the small intestine, with the edge of a Peyer's patch in the lower left hand corner. This image was acquired in situ, through the muscularis externa and serosa. All four tissue types are represented in this data set. (D) Image of the center of a Peyer's patch in the small intestine. A strong network of collagen and dense and evenly spaced lymphatic cells are visible. (E) A cancerous lesion in the small intestine of an APC*min*+/mouse. The epithelial structure is very different to that seen in normal tissues, and the presence of collagen is a feature of neoplasia. Scale bars are all 50 µm.

We used the blue and green emissions of the average uncalibrated versions of the basis spectra reported in Figure 3 to spectrally unmix five hyperspectral data sets acquired on fresh GI tissues as shown in Figure 4. Prior to unmixing, each basis spectrum was normalized to the maximum of its blue emission, and for display, each set of unmixed components was normalized to make the maximum of the epithelium image equal to 1. This means that the relative scale on each image represents the amount of each unmixed tissue type relative to the amount of epithelial tissue present. Figure 4A–C show normal tissues, A) at the glandular level of the colon, B) within the villi in the small intestine, and C) at the glandular level of the small intestine. Figure 4D shows the central part of a Peyer's patch, and Figure 4E shows a dysplastic lesion from the small intestine of an APC *Min*/+ mouse. Conventional RGB merges of these regions, acquired at 740 nm are shown in the left column, while the grayscale images shown in the other columns are images of the coefficients corresponding to the fit of each pixel to each of the four respective spectral signatures shown in Figure 3. Images that correspond to epithelial tissue components show either crypts (Figure 4A&C) or the epithelial tissue that covers the villi (Figure 4B). Collagen is represented strongly in the scaffold surrounding the glands of the colon tissue (Figure 4A), but not in the tissues from the small intestine (Figure 4B&C). Lymphatic tissue is present in all of the data sets, particularly in the Peyer's patch (Figure 4D), which is expected since it is a lymphoid aggregate. The Peyer's patch can also be seen to include almost no epithelial tissue and a significant network of collagen filaments. Changes associated with neoplasia are shown in Figure 4E. Abnormal epithelial overgrowth is apparent, as well as pockets of lymphatic tissue. The collagen image shows structure which is also different compared to the normal tissues, suggesting overproduction of extracellular matrix as is commonly associated with neoplasia [22]. Based on the possibility that our laminar propria basis spectrum may include porphyrin, it is interesting to note that the lamina propria components for all the samples reveal structures that correspond well to the likely locations of blood vessels (e.g. between crypts and in the center of villi). Noting this, we suspect that the disordered network revealed in the lamina propria image of the neoplastic lesion could correspond to excessive and abnormal vascularization within and around the tumor. These data are shown as false-color composite merges in Figure S3, along with representative H&E histology of these GI tissue types.

Overall, these hyperspectral unmixing results support the uniqueness of the basis spectra extracted from small representative regions of epithelium, laminar propria, collagen and lymphatic tissue, demonstrating that they are consistently able to identify these tissue types throughout a range of samples, mice and GI tissue types. The fact that the basis spectra chosen were averages from different samples to those that underwent unmixing further underscores the robustness of the signatures, as well as the promise of this approach for blind tissue delineation and identification.

It should be noted that for these hyperspectral unmixing results, data acquired in the red channel of our system was excluded. We chose to do this because, upon comparing our basis spectra to spectra sampled from a range of data sets, the shape of excitation spectra were found to match well, but the overall *relative amplitude* of the blue, green and red emission channels varied, with the red channel differing most significantly from the blue and green detection channels. Since we perform unmixing by concatenating the excitation spectra for the blue, green and red detection channels, we found that inclusion of the red channel often led to poor fits and large residuals. We believe that this effect may be due to the substantial and different levels of attenuation of visible (blue, green and red) light within tissue, such that images acquired at different depths, for example, would have different relative levels of attenuation for each emission channel. We particularly note that hemoglobin absorption, although large, is actually quite similar in the blue/green range, but dramatically different (much less) for the red emission range. Therefore, the presence of blood could be the cause of the relative amplitude variations between the detection channels that we have observed. Since most of the intrinsic fluorophores of interest emit in the lower blue-green spectral regions, and since the blue and green emission bands were found to vary, *relative to each other*, much less, we chose to exclude the red channel. However, we note that approaches such as allowing the relative amplitude of each emission channel to be a free parameter could feasibly have allowed inclusion of information from the red excitation spectrum.

This difficulty highlights the very significant advantage of spectral unmixing using *excitation*-scanning rather than using emission spectra; that in general, wavelength-dependent variations in tissue absorption and scattering over the near infrared range scanned during our hyperspectral acquisition (720–910 nm) are far less than in the visible range of 350–650 nm. Researchers acquiring spectral two-photon data consisting of only *emission* spectra (visible light) face many challenges compensating for the effects of depth and tissue-dependent variations in attenuation, and have not typically be able to perform spectral fitting or unmixing [23], [24], [25].

### Goodness of fit

**Figure 5:**
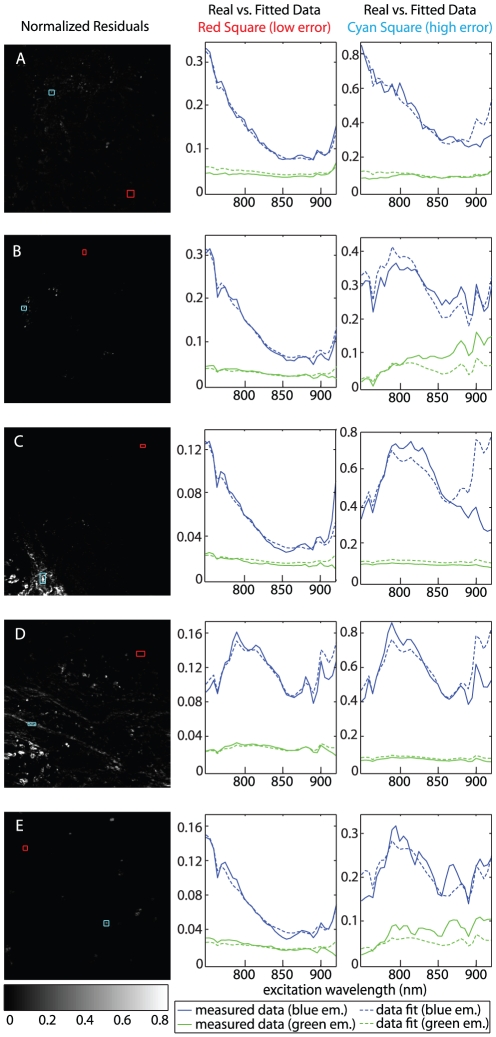
Residuals for the non-negative least squares fits of hyperspectral data that correspond to analysis shown in **Figure 4**. Images (left column) show the square of the Euclidean norm of the residuals from all excitation wavelengths. Plots (right) compare the measured spectra to the fitted data in selected areas of the images for a well matched region (red box) and a region with relatively high error (cyan box). (A) Glandular level in the colon. (B) Villi in the small intestine. (C) Glandular level of the small intestine. (D) Peyer's patch in the small intestine. (E) Lesion from the small intestine of an APC*min*/+ mouse.

The basis spectra used for the spectral unmixing described above were deliberately selected from data sets other than those that were unmixed, to test how well our approach can provide blind identification of tissue types. To quantify the performance of this hyperspectral unmixing strategy, we report the residuals of the non-negative least squares fits to our hyperspectral data in Figure 5. The value of each pixel in the images shown in Figure 5A–E represents the squared Euclidian norm of the residuals calculated for each excitation wavelength (the error output of the lsqnonneg function in Matlab™). We chose to present the error in this way because, as the sum of the squared residuals at each wavelength, the normalized residual represents the error for the entire fit across all excitation wavelengths and both emission bands. For each data set, we also compare measured spectra with corresponding spectral fits for regions of each image with high or low residual values. Selections outlined by red boxes in Figure 5 correspond to low error regions and show good agreement between the fit and the measured data in both the blue and green emission channels. Regions of the image with relatively high error, outlined by cyan boxes, can be seen to generally underestimate the real data for lower λ_ex_ and overestimate for higher λ_ex_, particularly in the blue emission channel. The error tends to be highest for structures that appear to be collagen. We suspect that the orientation dependence of SHG contributes to these errors, although these high residual regions could also imply that there is an additional spectral component (tissue constituent) that is not being accounted for by our four basis sets. Slight movement of the sample during a wavelength scan could also account for some of the fitting errors. Overall, however the fitting performance shown is fairly good, supporting our earlier conclusions that the extracted basis spectra well represent, and are capable of identifying the chosen basic tissue types within GI tissues.

## Discussion

We have introduced a new method for microscopic examination of fresh gastric mucosa. By using hyperspectral two-photon microscopy, we showed that 3D morphology can not only be visualized, but that different structures have unique spectral signatures. We demonstrated that these signatures can allow automated image segmentation, while also providing information regarding the identity of the fluorescence species composing different sub-tissue types.

Improved understanding of the intrinsic contrast of GI tissues, and the subcellular origins of specific spectral components, could provide guidance for enhanced wide-field endoscopic screening and imaging approaches [5], [6]. This is because our approach is able to not only delineate the different spectral components of these complex tissues, but assign them to their cellular-level origins and (in principle) quantify their likely contributions to bulk tissue spectroscopy measurements or wide-field fluorescence imaging. Furthermore, in future experiments we hope to explore the hyperspectral intrinsic contrast of *in-vivo* GI tissues in various stages of neoplasia, since there is substantial evidence that the metabolic and protein-binding state of tumor cells will exhibit distinctive spectral signatures to which hyperspectral two-photon microscopy could be sensitive [26], [27]. We expect that such microscopic hyperspectral studies could allow the design of more specific wide-field screening configurations that would target the key intrinsic spectral signatures of early disease.

Our results also demonstrate the power of non-linear microscopy and hyperspectral imaging for ‘instant histology’ or even insitu endoscopic microscopy [28]. The ability to rapidly evaluate fresh tissue biopsies for neoplastic changes at the bedside could reduce the cost and time associated with conventional histology, while also providing immediate diagnoses. Currently, two-photon imaging in human subjects is not FDA approved, due primarily to a lack of driver applications, few predicates, and the fear of light induced DNA damage. However, studies have shown that at low laser intensities, mutations are rare and therefore the future use of two-photon microscopes for human diagnostics is feasible [28], [29]. In fact, there have already been several demonstrations of its usefulness for diagnosis *in-vivo* in human skin [30]. For the case of GI diseases, screening and diagnosis generally requires endoscopy or colonoscopy, which often require lengthy procedure preparation and anesthesia. The ability to quickly determine whether suspicious lesions are malignant or benign could shorten or alter the course of the diagnostic procedure, as well as reducing the need for repeat colonoscopy if disease is found. Several groups have designed and implemented miniaturized two-photon probes for the purpose of endoscopic imaging [31], [32].

In addition to GI tissues, we have acquired and analyzed hyperspectral two-photon microscopy data on a wide range of other fresh tissues including kidney, retina, lung, ovary, heart, skin and spleen, all of which exhibited similarly exquisite contrast and structure resolvable without any tissue processing or use of contrast agents [33]. Consistent with other studies, our broader pilot measurements suggest much wider applications of hyperspectral two-photon microscopy beyond solely examining GI tissues [11], [34], [35]. We have even found that two-photon microscopy of intrinsic contrast can be used for examination of tissue specimens (e.g. clinical biopsies) that have been fixed and paraffin embedded. An example of fixed and embedded colon tissue imaging is included as supplemental data, which clearly shows 3D crypt structures and collagen SHG (Figure S4). The ability to image intact, but treated and even cryopreserved tissues could allow 3D visualization without destructive sectioning, as well as better planning for sectioning and staining [36]. Spectral analysis could also potentially provide beneficial supplementary chemical analysis of such tissue specimens, although we have noted that formalin fixed tissues have reduced variation in fluorescence as a function of excitation wavelength, consistent with degradation of contrast from substances such as NADH, FAD and porphyrins.

The main benefit of applying hyperspectral unmixing techniques to spectral two-photon data is that it captures more detail than looking at a single morphological image alone. For example, an image acquired at 740 nm can be examined to see that the endothelium is abnormal based on morphology, however, an image at 800 nm would need to be compared to appreciate the overlying collagen structure of this region. Inspecting the complete image stack could allow someone to appreciate a range of structures that come and go in different emission channels as excitation is varied. However, this would be painstaking and would require significant training and experience to do in reality. By applying unmixing strategies, we are effectively transforming this spectrally-encoded information into a ‘tissue-specific’ space, such that the unique spectral characteristics of each tissue are used to extract their morphology from the entire spectral data set. This is then analogous to specific *histological staining* of a particular species, with the substantial advantages that images can be acquired and analyzed rapidly and non-destructively, and can be visualized in multiple planes to appreciate structures spanning 200–400 microns in depth. The added benefit is that the extracted spectrum that is successful in unmixing a particular morphology correspondingly provides a type of in-situ chemical analysis of that structure through examination of the shape of its excitation and emission properties. Fit residuals rapidly demonstrate whether a fit is to be considered accurate, and visualizing residual maps and spectra as shown in Figure 5 can readily provide guidance for the selection of new basis spectra from additional regions that may represent a previously unaccounted for species or tissue type within the sample. In turn, these new spectra can be incorporated into another fit and used to map the locations of this new species, and the spectra can be examined to determine the likely identity of the anomalous component.

Finally, most spectral two-photon studies are carried out by only analyzing emission spectra [23], [24], [25]. Such emission spectra can be challenging to acquire, adding significant complexity to two-photon instrumentation and often resulting in poor signal to noise. In contrast, excitation-based spectral data can be acquired with relative ease using automatically tunable lasers (such as the Spectra Physics MaiTai series, or Coherent Chameleon series). However, the real advantage of using two-photon excitation spectra for unmixing, rather than emission spectra, is that for a given emission channel, there is very little variation in the attenuation of excitation light scanning from 710–920 nm light. Over the visible range corresponding to emission-side spectral analysis (350–650 nm), the scattering properties of tissue vary substantially, and a range of absorbers, particularly hemoglobin, have strong and distinct wavelength-dependent absorption variations [37]. The kinds of variability that we see between the absolute signal intensity in our red, green and blue detection channels for different tissues and depths underscores the difficulties faced by those attempting to perform spectral unmixing or fitting on emission spectra from two-photon microscopes compared to hyperspectral unmixing using excitation scanning.

## Supporting Information

Figure S1 **Mean spectral signatures of the four tissue components before FAD calibration.** Error bars are one standard deviation above and below each data point. The excitation wavelength dependent envelope is present in all data sets A.–D. Calibration to FAD was done in order to better identify the possible sources of contrast and to prevent confusion in interpreting these spectral signatures. The FAD spectrum used for calibration is included in Figure S2.
(EPS)

Figure S2 **FAD spectral calibration, and corrected spectra of in-vitro pure fluorophore samples.** Shows raw excitation spectra for three emission bands (red, green and blue) acquired on a sample of pure FAD (a 400 µM solution in phosphate buffered saline (PBS), Sigma Aldrich F6625). The excitation wavelength-dependent envelope (given by K in equations 1 and 2) is present in all data sets and is caused by a range of multiplicative systematic effects including wavelength-dependent variations in dispersion. One-photon measurements of FAD suggest that its excitation spectrum is fairly flat across this excitation range (Radosevich et al, Optics Letters 2008), such that it can be assumed to provide a measure that is proportional to K. All ‘corrected’ spectra shown are therefore wavelength-calibrated by dividing them by the excitation spectrum (for the green emission) acquired on this pure FAD sample. Center; an uncorrected excitation spectrum of a pure sample of NADH (400 µM solution in PBS, Sigma Aldrich N6636), and Right; the corrected spectrum after dividing all three emission channels (red, green and blue) by the green-emission excitation spectrum of FAD. B. Shows wavelength-calibrated spectra from a 300 µM solution of Zinc-protoporphyrin IX (Sigma Aldrich P8293) dissolved in dimethylsulfoxide (DMSO). C. Shows wavelength-calibrated spectra of phospholipid mixture dissolved in chloroform (Sigma Aldrich P3817). All in-vitro samples were measured in a small reservoir topped with a coverslip. D. Shows the excitation spectra extracted from the center of a large blood vessel imaged in the living brain of a rat as shown in E. No exogenous contrast is present in the image, and the blood vessel can be clearly discerned, making it clear that the red fluorescence signal observed is coming from whole blood. All error bars show one standard deviation above and below the mean.
(EPS)

Figure S3 **False color merges of component images.** In (A), component images are shown with false color with the merge at right. The color scheme used is consistent for all the unmixed images in B–F, with yellow corresponding to epithelium, white to lamina propria, blue to collagen, and green to lymphocytes. Scale bars for all images are 50 µm. (B) At the crypt level of colon, the epithelial tissue is surrounded by a mixture of the three other components: lamina propria, collagen, and lymphatic tissue. Corresponding histology (G) shows connective tissue around the crypts (black arrow) as well as a few dark staining lymphatic cells (red arrow). (C) In the small intestine, the main components of the cores of the villi are lymphatic tissue and lamina propria. Histology (H) shows epithelium surrounding each villus and loose connective tissue forming the core of the villi. (D) At the boundary of a Peyer's patch and the crypt level of small intestine, all four components are visible. Lamina propria signal is particularly strong along the blood vessel at the boundary (arrow) (I) Histology of the Peyer's patch boundary. (E) Close up of the lymphatic cells that comprise a Peyer's patch. The strong signal from lymphatic tissue and collagen ‘co-stain’ this false color merge. Peyer's patch histology (J) shows the darkly staining nuclei of lymphatic cells, but without special staining cannot identify the connective tissue. (F) Merge shows the spatial registration of the four components in a neoplastic region of an APC*min*/+ mouse. Histology of a lesion (K) shows a thickened and irregular epithelium which is characteristic of neoplasia.
(TIF)

Figure S4 **Intrinsic contrast two-photon microscopy image of an unstained, fixed, paraffin embedded colon tissue sample.** Images were acquired at an excitation wavelength of 850 nm, at various depths within the paraffin block as indicated by the z value in the upper right corner of each image. Cellular level structures can be clearly seen, along with collagen SHG (blue in images A–C). Scattering appears to have a greater effect on depth penetration in images D–F than in fresh tissues acquired at the same depth, but the morphology of the embedded tissue is visible. Scale bar is 50 µm.
(EPS)

Video S1 **Axial stack of the villi in the small intestine.** This movie displays the morphology of a few villi with cellular resolution from the lumen to a depth of 100 µm. Stack was acquired at 740 nm and the field of view is 375 µm on each side.
(WMV)

Video S2 **Axial stack of the small intestine beginning at the outer muscularis mucosa.** Imaging the small intestine through the muscularis mucosa allows better visualization of the crypts in the lamina propria. This stack was acquired at 740 nm with a field of view of 375 µm on each side and a total depth of 170 µm. Note that at z = −115 µm, the villi begin to form. Villi are visible at z = −150 µm.
(WMV)

Video S3 **Wavelength scan of glandular level in the small intestine**. This movie shows the changes in emission that result from varying the excitation wavelength from 710 nm to 920 nm in 5 nm increments. The excitation wavelength of each image step is shown in the upper left corner.
(WMV)
