# Supplementary figures and images for: Spectral Characterization and Unmixing of Intrinsic Contrast in Intact Normal and Diseased Gastric Tissues Using Hyperspectral Two-Photon Microscopy
Source: PLoS One. 2011 May 16;6(5):e19925. doi: 10.1371/journal.pone.0019925 (PMC3095627; doi:10.1371/journal.pone.0019925)

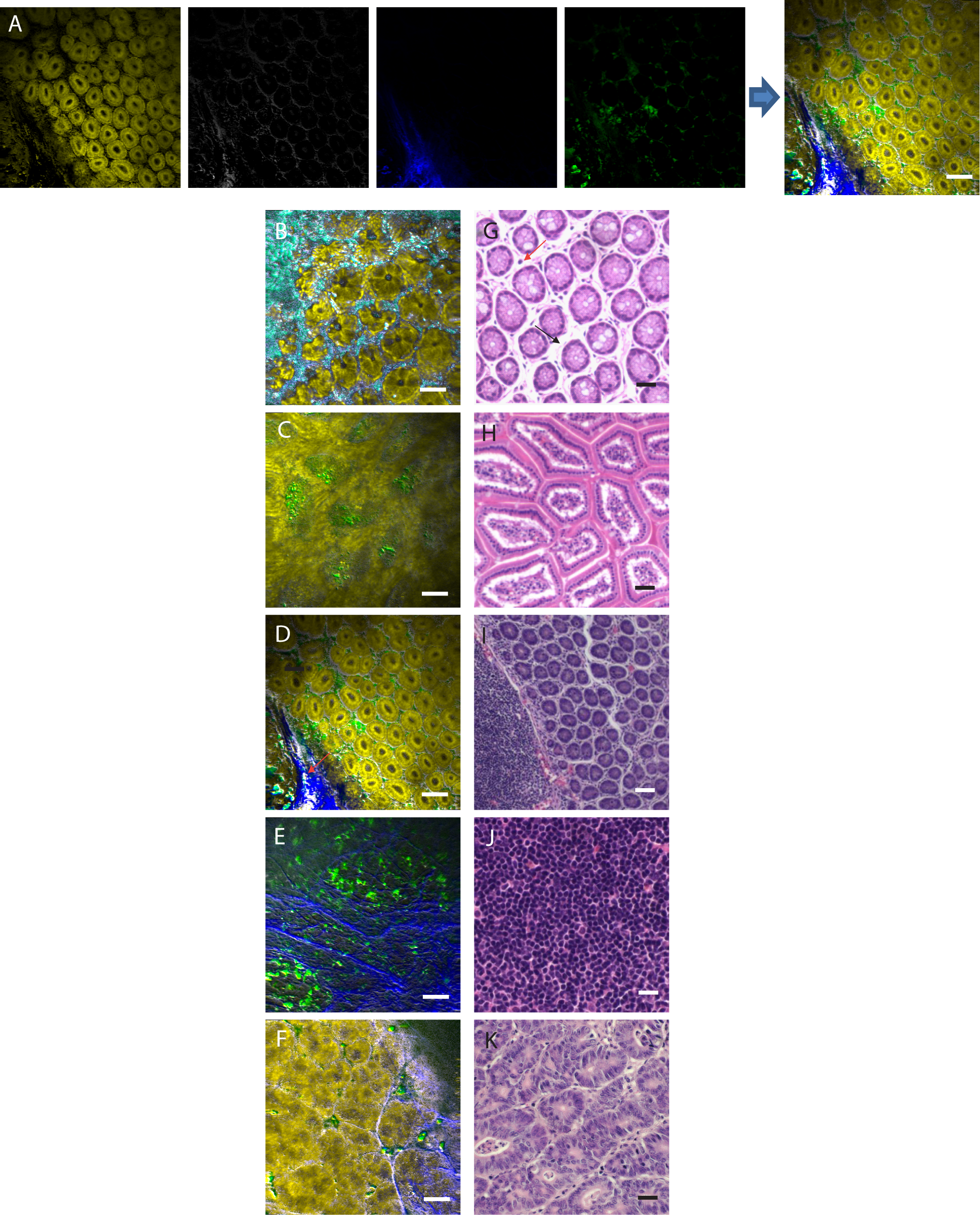

Supplement: Figure S3 — False color merges of component images. In (A), component images are shown with false color with the merge at right. The color scheme used is consistent for all the unmixed images in B–F, with yellow corresponding to epithelium, white to lamina propria, blue to collagen, and green to lymphocytes. Scale bars for all images are 50 µm. (B) At the crypt level of colon, the epithelial tissue is surrounded by a mixture of the three other components: lamina propria, collagen, and lymphatic tissue. Corresponding histology (G) shows connective tissue around the crypts (black arrow) as well as a few dark staining lymphatic cells (red arrow). (C) In the small intestine, the main components of the cores of the villi are lymphatic tissue and lamina propria. Histology (H) shows epithelium surrounding each villus and loose connective tissue forming the core of the villi. (D) At the boundary of a Peyer's patch and the crypt level of small intestine, all four components are visible. Lamina propria signal is particularly strong along the blood vessel at the boundary (arrow) (I) Histology of the Peyer's patch boundary. (E) Close up of the lymphatic cells that comprise a Peyer's patch. The strong signal from lymphatic tissue and collagen ‘co-stain’ this false color merge. Peyer's patch histology (J) shows the darkly staining nuclei of lymphatic cells, but without special staining cannot identify the connective tissue. (F) Merge shows the spatial registration of the four components in a neoplastic region of an APCmin/+ mouse. Histology of a lesion (K) shows a thickened and irregular epithelium which is characteristic of neoplasia. (TIF) [file pone.0019925.s003.tif]
